# Industrial Internet of Things Gateway with OPC UA Based on Sitara AM335X with ModbusE Acquisition Cycle Performance Analysis

**DOI:** 10.3390/s24072072

**Published:** 2024-03-24

**Authors:** Cornel Ventuneac, Vasile Gheorghita Gaitan

**Affiliations:** 1 Faculty of Electrical Engineering and Computer Science, Stefan cel Mare University, 720229 Suceava, Romania; 2 Integrated Center for Research, Development and Innovation in Advanced Materials, Nanotechnologies and Distributed Systems for Fabrication and Control (MANSiD), Stefan cel Mare University, 720229 Suceava, Romania

**Keywords:** PRU, ModbusE, System on Chip, acquisition cycle, OPC UA, IIoT gateway

## Abstract

This article presents the hardware and software architectures used to implement the Modbus Extension (ModbusE) IIoT gateway, the performance of the acquisition cycle at the PRU real-time programmable core level, the acquisition cycle communication flow—dispatcher—OPC UA server (Linux)—OPC UA client (Windows) as well as the performance analysis of data communications between the IIoT ModbusE gateway and the OPC UA client (Windows). In order to be able to implement both the ModbusE acquisition cycle and the OPC UA server, the BeagleBone Black Board was chosen as the hardware platform. This board uses the Sitara AM335x processor (Texas Instruments (TI), Dallas, TX, USA) from Texas Instruments. Thus, the acquisition cycle was implemented on the PRU0 real-time core, and the OPC UA server, running under the Linux operating system, was implemented on the ARM Cortex A8 processor. From the analysis of the communication speed of the messages between the OPC UA client and the ModbusE servers, it was found that the ModbusE acquisition cycle speed was higher than the acquisition speed of the OPC UA client.

## 1. Introduction

Nowadays, local industrial networks are indispensable in application areas such as the machine building industry, building automation and construction, factory automation, aeronautics, energy distribution, industrial process control, etc. In general, industrial process automation is achieved using more sensors and execution elements, which at the same time leads to an increase in clocking systems in the production process. Connections between all equipment in the production process are made by means of connecting wires. Due to falling prices for process systems and rising prices for connecting wires, the time becomes clear for serial process control if input and output modules are located close to the sensors and adapters. Local industrial networks should be seen as an integrated part of automation processes and not as stand-alone solutions. Networks for the lowest levels of the automation hierarchy or so-called fieldbus systems have led to the increased flexibility and performance of automation systems. Given the large size of automation systems and installations, the benefits of fieldbus systems are clear. A more general definition for fieldbus is given by the Fieldbus Foundation as follows [1]: fieldbus is a digital, bi-directional communication between intelligent measurement and control devices. It represents a local area network (LAN) for industrial process control, automation applications, etc. The Ethernet as a local area network (LAN) is becoming increasingly used in automation, but it seems that an industrial Ethernet does not make the fieldbus levels completely obsolete. Fieldbuses are better optimized for specific automation process attributes than general Ethernet networks. Fieldbus represents a step towards decentralization and increasing the quality of the control process [2]. Also, other advantages of the fieldbus concept are its modularity, the possibility of expanding the installations and the ability to have intelligent devices that can communicate both for data transfer and maintenance and configuration [3,4]. A different design approach was to consider the network systems within the process control as columns of real-time distribution systems [5]. As a result, the distinction between LAN (local area network) and FAN (field area network) should be based on the functionality and scope of these networks. So, FAN is a network used in automation processes regardless of the topology, protocol or real-time requirements.

The IIoT (Industrial Internet of Things) is usually used in manufacturing processes and refers to the industrial subset of the Internet.

In recent years, concerns in the field of industrial local area networks have grown so that a plethora of specifications and communication protocols have emerged, and the efforts of specialists in this field are focused on standardization and a reduction in the number of standards. In local area networks (LANs), the connection between intelligent sensors and execution elements is performed using communication protocols (Modbus, Profibus, CANOpen, Lonwork, MQTT, etc.).

Some protocols are simple and straightforward, while others are complex and require more sophisticated hardware resources. Modicon in 1979 made RS232 serial communication possible for PLCs produced by their company. The Modbus protocol is a unique protocol and is one of the most popular protocols among automation devices. In paper [6], the authors present an original ModbusE extension, which is intended to extend the classical Modbus specification by introducing a time variable and, thus, transforming the Modbus protocol into a fully defined protocol. Unlike the Modbus classic, ModbusE defines an acquisition cycle (AC) that allows the periodic acquisition of values from the server stations at well-determined time intervals. Moreover, within the AC, the values transmitted on the ModbusE network can be seen as being in a publisher-subscriber architecture. In ModbusE, at the level of one slot, minus slots 0 and 1, messages can be sent that are fully compatible with Modbus classic. In the data area of a slot, specific messages can be sent to other protocols such as CANopen, PROFIBUS, etc. Using gateway servers, connections can be made with the resources at the ModbusE gateway level (such as Modbus TCP/IP, OPC UA), which allows the connection of messages from other protocols on the Internet (IIoT). The data area of the ModbusE message can be defined as carrying only the valuable part of classic Modbus messages, thus increasing the bandwidth of the communication channel. The data area of the ModbusE message can be used according to the application’s requirements. In paper [7], the authors present a gateway based on Modbus/MQTT using Raspberry Pi that can be used in industrial cloud IoT applications. In paper [8], the authors present a monitoring solution for smart buildings using OPC UA. In papers [9,10], the authors present the experiments and results obtained for the implementation of the acquisition cycle with ARM Cortex M0, M4 and M7 architectures. In paper [11], the authors present a Modbus gateway for highly reliable communication where every controller can act as a master to start communication with devices on the virtual Modbus RTU network. In paper [12], the authors present a controller using the STM32 microcontroller for a universal testing machine based on Modbus TCP. In Ref. [13], the authors present MUbus as a SCADA protocol based on the Modbus protocol. In paper [14], the authors present Modbus TCP and Modbus RTU to implement a double-layer monitoring network. In paper [15], the authors describe an energy monitoring system based on the Modbus protocol and present the tests using 250 W and 500 W resistive loads.

The contributions made in this paper are the hardware and software architectures used to implement the ModbusE IIoT gateway on Sitara AM335x. The Sitara AM335x offers two real-time PRU cores that allow the implementation of the Modbus Extension and a Cortex A8 processor for the implementation of an OPC UA server, which is useful for IIoT applications. The present work presents a complete IIoT solution that, in addition to the ModbusE acquisition cycle, also presents an integrated OPC UA server on a single Sitara AM335x microcontroller (Texas Instruments (TI), Dallas, TX, USA) architecture. In the architectures previously made with ModusE, communication with the higher levels was performed with Modbus TCP/IP.

The remainder of this paper is organized as follows: Section 2 describes the hardware and software architectures of the IIoT gateway. Section 3 describes PRU0 (Programmable Real-Time Unit) performance. Section 4 describes the communication channel modeling of the acquisition cycle. Section 5 describes the flow of communication messages between the OPC UA client and IIoT gateway, and Section 6 describes the performance analysis of the IIoT gateway data communications and the OPC UA client.

## 2. IIoT Gateway Hardware and Software Architectures and Networking

In paper [10], the authors show that by using Cortex Mx architectures at a performance of approximately 49.6%, useful data from the acquisition cycle can be obtained with STM32F407 at a speed of 10.5 Mb/s on the serial port, with 36% useful data from the acquisition cycle with STM32F746 at a speed of 27 Mb/s on serial port and 58.9% useful data from AC with LPC4357 which has 2 Cortex M0 and Cortex M4 cores at 11.5 Mb speed/s on the serial port. In this paper, we started from the results obtained by the authors in paper [10] and present a solution using the Sitara AM335x processor from Texas Instruments on the Beaglebone Black system. The presence of PRU (Programmable Real-Time Unit) processors leads to better performance on the physical channel data flow, and, at the same time, due to the ARM Cortex A8 processor, there is support for implementing IIoT connections based on OPC UA. As presented in paper [16], the Sitara AM335x processor from Texas Instruments was chosen for the implementation of the IIoT gateway. The SitaraAm335x consists of a 32-bit ARM Cortex A8 RISC processor operating at a frequency of 1 GHz and two 32-bit PRU (Programmable Real-Time Unit) cores operating at a frequency of up to 200 MHz. One of the two PRU cores (PRU0) helped to improve data channel utilization, and the ARM Cortex A8 processor helped to provide a high-performance IIoT connection. The PRU core allows easy implementation of the ModbusE protocol at 12 Mb/s, while the ARM Cortex A8 processor with the Linux operating system supports the implementation of an OPC UA server with different middleware communication modes on the same system. The old versions with ARM Cortex Mx architectures used Modbus TCP/IP for Internet communication. So, the solution with Sitara AM335x is much more closely integrated by offering the OPC UA server. The solution on Sitara AM335x can also be used on other architectures, such as the one presented in this work [17].

**Figure 1:**
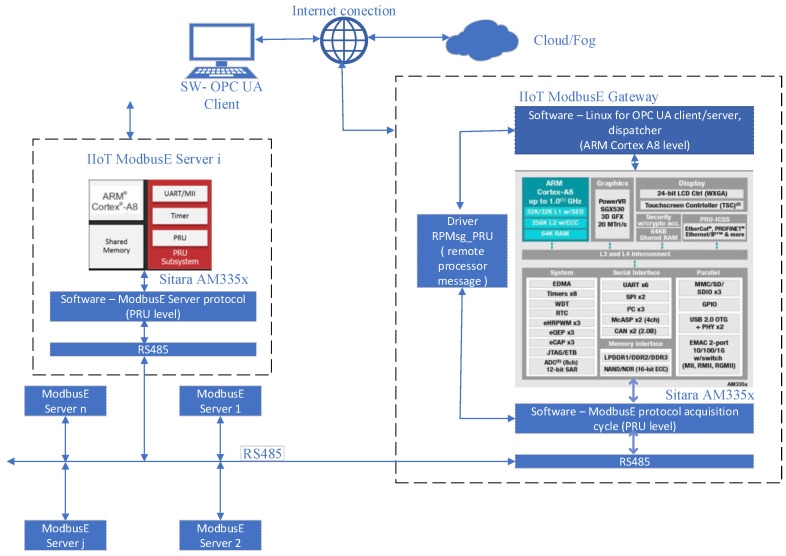
Main experimental system architecture for IIoT gateway—ModbusE.

Figure 1 shows the main architecture of the experimental IIoT—ModbusE gateway system. The IIoT ModbusE Gateway module in Figure 1 represents the master (client) station, also called the BSG (Base Station Gateway). This BSG implements the acquisition cycle (AC) at the PRU0 core, the OPC UA server and client application, as well as the dispatcher application at the ARM Cortex A8 processor. The OPC UA server and client application takes commands from an OPC UA client (running on a Windows operating system computer) via TCP/IP and forwards them to the dispatcher application. The dispatcher application takes commands from the OPC UA server and client application and sends them on to the purchasing cycle (CA). The dispatcher takes the response to commands from the acquisition cycle and sends it on to the OPC UA server and client application. The OPC UA server and client application, in turn, forward the response to the OPC UA client via TCP/IP. The IIoT ModbusE Server module in Figure 1 represents the slave station (server), which was also implemented on the Sitara AM335x processor. This station takes data from sensors or sends commands to execution elements or actuators following commands from the BSG station. The other stations (ModbusE Server1, …, ModbusE Server n) represent other workstations using the ModbusE extension but are implemented with other technologies. The slave (server) stations communicate with the ModbusE Gateway IIoT module via serial ports using the RS485 serial line standard. The performance analysis of the IIoT—ModbusE Gateway experimental system is conducted for an acquisition cycle consisting of 10 slots.

## 3. PRU Performance

As presented in Chapter II of this paper, the PRU0 core on the Sitara AM335x processor was chosen to implement the Base Station Gateway (BSG) acquisition cycle (AC). This core is a Programmable Real-Time Unit (PRU) and operates at a frequency up to 200 MHz. Communication between the BSG station and the slave (server) stations is via the PRU UART serial port. The PRU UART port operates at speeds of up to 12 Mbps, so the duration of the 10-bit character to be processed is 0.83 microseconds. Measurements show that the duration of one bit at 12 Mbps is approximately 83.19 ns, and the period of one acquisition cycle with 10 slots is 1.349 ms. So, it follows that the maximum possible number of bits per acquisition cycle is 16,210 (1349 μs/0.08319 μs). Theoretically, a number of 1621 10-bit characters can be transmitted under continuous transmission conditions per the 10-slot acquisition cycle. But in reality, 1137 characters (3 + 4 + 20 + 40 + 80 + 96 + 510 + 128 + 128 + 128—see Section 4 for more details) were transmitted per 10-slot acquisition cycle in the experimental system. This gives a total message (start address, start and stop bits, CRC checksum) of 70.1% (11370/16210 = 0.701). Subtracting the start address characters and the CRC checksum characters from the number of characters actually sent per acquisition cycle (with 10 slots transmitted and 8 slots received, as slot 0 and slot 1 do not receive characters) gives a total of 1082 characters per acquisition cycle and, thus, a useful message of 66.7% (1082/1621 = 0.667). A payload of 53.3% ((1082 × 8)/(1621 × 10) = 0.533) is obtained. The software application that implements the acquisition cycle on the PRU0 core (of the PRU-ICSS subsystem) for the master station (client) uses the status and control bits of the UART (Universal Asynchronous Receiver/Transmitter) and IEP (Industrial Ethernet Peripheral) peripheral ports. The IEP timer is used with the following two comparators: one comparator is used to determine the end of a message on reception, and the second comparator is used to signal the end of a slot duration. When the end of the slot duration is signaled, the comparator register is loaded with the duration of the next slot, and the RS485 driver is switched to transmit, after which the message transmission is started using the empty transmit register flag. During this time, the overflow indicator for the slot duration is also tested. If an overrun has occurred, the slot message transmission error is signaled, the driver is switched to receive, and the next slot is proceeded to. If the whole message has been sent, the driver is switched to receive and waits for the first character or overrun to be received for the slot duration, in which case a transmission error is signaled; the driver is switched to receive and moves to the next slot. If a character is received, the driver stays on receive until overrun is indicated for the end of the message on receive. Also, in this loop, the slot duration overrun indicator is tested, and the same actions as above are taken. Then, the driver switches to transmit and waits for the slot duration end indicator to pass before resuming the main loop with the next slot (slot_next = slot% NrMaxSlot). The software for the server (slave) station is implemented in a similar way, with the difference at the server (slave) level being the operation of moving the message from the receive buffer to where the application buffer occurs. When testing on the experimental system with a 10-slot acquisition cycle, the time durations for the slots are calculated mathematically. But, with these values for the slot durations, the acquisition cycle does not start working properly. For the acquisition cycle to start working properly, it is concluded that the slot durations should be adjusted with an adjustment time tAdjust. This tAdjust is determined empirically for each slot. It includes some communication times between the hardware components of the Sitara AM335x processor that are difficult to highlight and adjust. In the future, we foresee the creation of software for the automatic generation of these times. This tAdjust achieves the adjustment of the acquisition cycle so that it does not cause errors. The time period between transmission and reception for a slot with 510 characters, for example, depends on the processing times of the slave (server) stations, such as the time to calculate the CRC sum to check the correctness of the received message, the time to move the message from the reception buffer to the execution task buffer if the received request is to write to the slave, and the time needed to form the response if the received request is to read from the slave station. These times are more difficult to know and are retrieved in the tAdjust time. When the master station (client) receives the reply, a time for the CRC sum calculation occurs to check the integrity of the message received from the slave station.

## 4. Communication Channel Modeling of the Acquisition Cycle

The acquisition cycle within the ModbusE extension consists of slots. Each slot has a data structure with status, control and data information. The minimum number of slots in an acquisition cycle is three. The ModbusE extension uses the following two types of objects: the PDO (Process Data Object), which is used for process data transfer and the SDO (Service Data Object), used for configuration, maintenance and testing [9,18]. These objects (PDO and SDO) can be transported over the network using messages (multiple messages make up transactions). This ModbusE extension has three types of messages as follows: SDA (Send Data with Acknowledge), SDN (Send Data with No Acknowledge), and SRD (Send and Request Data) [9,18].

A frame consists of the following:

1f = 1 Start bit + 8 data bits + 1 Stop bit = 2HT + 8 data bits = 10 bits(1)

where f—frame and HT—header trailer.

A slot consists of the following:

1 slot = 1 write message + 1 read message.(2)

1 message = 1 frameheader (address slot) + ni f(date) + 2f(CRC) = 3f(HT) + ni f(date)(3)

We used an acquisition cycle with 10 slots.

Slot 0 = 1 write message = 3f(HT);(4)

Slot 1 = 1 write message = 3f(HT) + 1 control character;(5)

Slot 2 = 1 write message + 1 read message = (3f(HT) + 13f(date)) + (3f(HT) + 1f(date));(6)

Slot 3 = 1 write message + 1 read message = (3f(HT) + 29f(date)) + (3f(HT) + 5f(date));(7)

Slot 4 = 1 write message + 1 read message = (3f(HT) + 61f(date)) + (3f(HT) + 13f(date));(8)

Slot 5 = 1 write message + 1 read message = (3f(HT) + 61f(date)) + (3f(HT) + 29f(date));(9)

Slot 6 = 1 write message + 1 read message = (3f(HT) + 252f(date)) +(3f(HT) +252f(date));(10)

Slot 7 = 1 write message + 1 read message = (3f(HT) + 61f(date)) + (3f(HT) + 61f(date));(11)

Slot 8 = 1 write message + 1 read message = (3f(HT) + 61f(date)) + (3f(HT) + 61f(date));(12)

Slot 9 = 1 write message + 1 read message = (3f(HT) + 61f(date)) + (3f(HT) + 61f(date));(13)

NfAC—number of frames per acquisition cycle.

NfAC = 3f + 4f + 20f + 40f + 80f + 96f + 510f + 128f + 128f + 128f = 1137f.(14)

Max → 11,370 b/AC.

We used 1 bit at 12 Mb/s → 83.19 ns from the calculation at 83.33 ns.

The period of a 10-slot acquisition cycle is 1.349 ms

1349/0.08319 = 16,210 b/AC(15)

From the division,

11,370/16,210 = 0.701(16)

resulting in a total message of 70.1% (with the start address, start and stop bits and the CRC count sum).

If the number of characters to be transmitted per 10 m slot acquisition cycle is reduced by the characters for the start address and checksum,

1137 − 55 = 1082(17)

where 55 represents the characters for the start address and CRC checksum for 10 transmitted slots, and 8 received slots per acquisition cycle plus 1 control character for slot 1.

From the division,

1082/1621 = 0.667(18)

resulting in a useful message of 66.7%.

From the formula,

(1082 × 8)/(1621 × 10) = 0.533 (the start and stop bits were removed)(19)

resulting in a 53.3% payload (no start address, CRC checksum, start and stop bits or characters were included that were not useful data).

## 5. Flow of Communication Messages between Acquisition Cycle (on PRU0), Dispathcer (on ARM Cortex A8 with Linux), OPC UA Server/Client (on ARM Cortex A8 with Linux), and OPC UA Client (on Windows)

**Figure 2:**
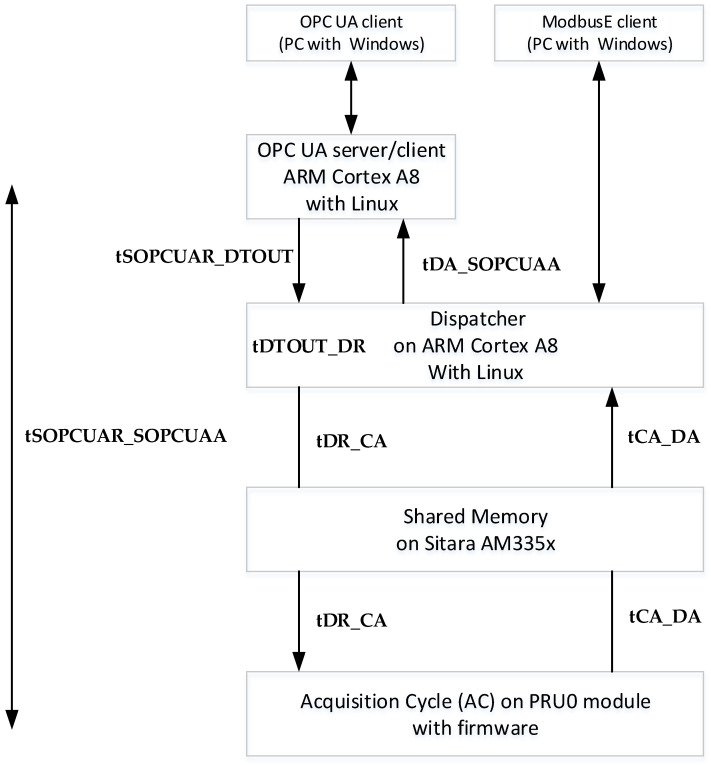
Flow of messages communication between the OPC UA/ModbusE client, dispatcher and acquisition cycle (AC).

As can be seen in Figure 1, the ARM Cortex A8 processor can run the OPC UA server and client. For this, we ported open62541 to the ARM Cortex A8 processor. The open62541 library is open source and is a free implementation of the OPC UA protocol written in the C++ programming language. The OPC UA server running on the ARM Cortex A8 processor is called ServerOPCUAModBusE. The ServerOPCUAModBusE application is used as a client application for communication with the BBB_ARM_A8_MBE_IOT_GATEWAY communication server (dispatcher). Communication is performed via sockets. The ServerOPCUAModBusE application is used as the server application for communication with the OPC UA client (with the OPC_UA client running on a computer using Windows as the operating system). Figure 2 shows the flow of communication messages between the OPC UA/ModbusE client (on Windows), the dispatcher (on ARM Cortex A8), server OPC UA (on ARM Cortex A8) and the acquisition cycle (on PRU0). Therefore, the OPC UA client that runs on a PC with Windows as the operating system sends ModbusE commands via TCP/IP sockets to the OPC UA server that runs on the ARM Cortex A8 processor (Linux). The OPC UA server sends the MosbusE commands to the dispatcher via TCP/IP sockets. The purpose of the dispatcher is to interpret and mediate MobusE commands (messages) between the OPC UA server or ModubusE clients and the acquisition cycle. Then, the dispatcher sends ModbusE commands to the acquisition cycle via shared memory. The acquisition cycle disables the slot at which the command was received (so that the acquisition cycle does not overwrite the memory area for the response to the command). The acquisition cycle then notifies the dispatcher that the command was received and responds to the ModbusE command. When the dispatcher receives the response for the ModbusE command from the acquisition cycle, it activates the slot from which it received the answer and sends this response either to ModbusE clients (if any did send a command) or to the OPC UA server. The OPC UA server sends the response to the OPC UA client that runs on a PC. The OPC UA client used is Unified Automation’s UaExpert and runs on a Windows operating system, as mentioned above. In the address space of the ServerOPCUAModBusE server, we entered two String variables for the ModBusE command and the ModBusE response.

The times presented in Figure 2 are as follows:

- tSOPCUAR_DTOUT—the time between the server OPC UA’s request and timeout on the dispatcher.
- tDTOUT_DR—the time between the dispatcher’s timeout and the dispatcher’s request to the acquisition cycle.
- tDR_CA—the time between the dispatcher’s request and the acquisition cycle’s reception of the command.
- tCA_DA—the time between the acquisition cycle’s response and the dispatcher’s reception of the command answer.
- tDA_SOPCUAA—the time elapsed between the dispatcher sending the answer of the command and server OPC UA reception of the answer.
- tSOPCUAR_SOPCUAA—the time between the server OPC UA request and server OPC UA received the answer.
- tSOPCUAR_SOPCUAR—the time between two consecutive server OPC UA requests.

The OPC client runs on a computer with Windows as the operating system. In the OPC client, we had two objects of the String type as follows:

- Command ModBusE, where the user enters the ModbusE command and presses enter.
- Response ModBusE, where the user receives the answer to the command.

## 6. Performance Analysis of IIoT Gateway Data Communications, OPC UA Client (Windows)

In this chapter, we start with the analysis of the time required for the information requested by the OPC UA server (ServerOPCUAModBusE) running on the ARM Cortex A8 processor (under the Linux operating system) to traverse the following path: OPC UA server (ServerOPCUAModBusE)—dispatcher (BBB_ARM_A8_MBE_IOT_GATEWAY—also running on the ARM Cortex A8 processor)—acquisition cycle (with CA running on the PRU0 real-time kernel)—dispatcher—OPC UA server.

**Figure 3:**
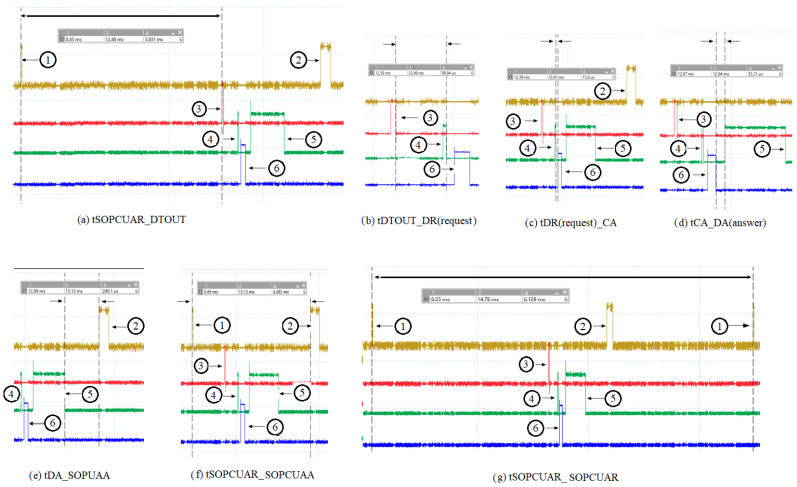
Measured times required for the information requested by the OPC UA server to reach the acquisition cycle and return.

In Figure 3, the numbered spikes represent the following: 1 represents the launch of the command by the OPC UA server running on the ARM Cortex A8 processor; 2 represents the response of the command received by the OPC UA server relaying on the ARM Cortex A8 processor; 3 represents the time when the dispatcher running on the ARM Cortex A8 processor tests if there is a command from the OPC UA server; 4 represents the time when the dispatcher sends the command to the acquisition cycle (CA—running on the PRU0 core); 5 represents the moment when the dispatcher (after the acquisition cycle has executed the received command) has finished the read or write operation in the shared data area (slot data without the acquisition cycle) and has reactivated the slot from which the command was sent; and 6 represents the moment when the request from the dispatcher is taken over by the acquisition cycle (CA) and the slot from which the request was sent is deactivated. The time tSOPCUAR_DTOUT (between spike 1 and spike 3) represents the time between the moment the OPC UA server (ServerOPCUAModBusE—running on the ARM Cortex A8 processor) sent the request to the dispatcher (BBB_ARM_A8_MBE_IOT_GATEWAY—running on the ARM Cortex A8 processor) and the moment the dispatcher tests for a request from the OPC UA server. The time tDTOUT_DR(request) [between spike 3 and spike 4] represents the time between the time the dispatcher tested if there was a request from the OPC UA server and the time it sent the request further to the acquisition cycle. The time tDR(request)_CA (between spike 4 and spike 6) is the length of time between when the dispatcher sent the request to the acquisition cycle (CA) and when the acquisition cycle retrieved the request from the dispatcher. The time tCA_DA(answer) [between spike 5 and spike 6] represents the time between the moment when the acquisition cycle retrieved the request from the dispatcher, deactivated the slot from which the request came in, executed the request and the moment when the dispatcher completed the read or write operation in the shared data area (the slot data of the acquisition cycle) and reactivated the slot from which the request was sent. The time tDA_SOPUAA (between spike 2 and spike 5) is the time between when the dispatcher, after preparing the response for the request, sent the response to the OPC UA server and the time when the OPC UA server retrieved the response from the dispatcher. The time tSOPCUAR_SOPCUAA (between spike 1 and spike 2) is the duration between the OPC UA server sending the request (command) to the dispatcher and the server receiving the request response from the dispatcher. Time tSOPCUAR_SOPCUAR (between spike 1 and spike 1) is the time between the OPC UA server launching a request to the dispatcher and the time the OPC UA server launches the next request to the dispatcher (the time between two consecutive requests).

Table 1 presents the values of the times shown above for 10 oscilloscope measurements.

**Figure 4:**
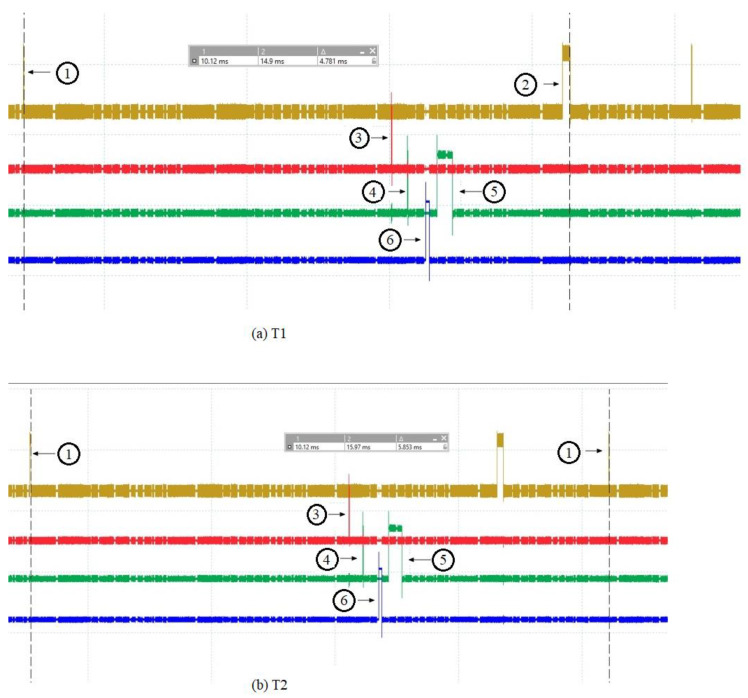
Measured time required for the information received on server OPC UA from the OPC UA client to reach the acquisition cycle and return (**a**) and measured time for two consecutive requests from the OPC UA client (**b**).

In Figure 4, the numbered spikes represent the following: 1 represents when the OPC UA server (running on the ARM Cortex A8 processor) receives the request via sockets from the OPC UA client (running on a computer using the Windows operating system) and prepares to send it on via sockets to the dispatcher; 2 represents when the OPC UA server receives the request response from the dispatcher and the numbered spikes 3, 4, 5 and 6 are the same as in Figure 3 and, therefore, are described in Figure 3. Time T1 is the time between the OPC UA server preparing to forward the request from the OPC UA client to the dispatcher and the OPC UA server receiving the response from the dispatcher and preparing to forward it to the OPC UA client. Time T2 is the time between two consecutive requests that the OPC UA server prepares to make.

In Table 2, the values for the times T1 and T2 described above are shown through 10 oscilloscope measurements.

## 7. Discussion

Previous work using the ModbusE extension on Cortex Mx architectures has achieved a performance of around 49.6% useful data at a 10.5 Mb/s serial port speed with STM32F407, 36% useful data at a 27 Mb/s serial port with STM32F746 and 58.9% useful data at 11.5 Mb/s serial port speed with LPC4357 which has 2 Cortex M0 and M4. But Cortex M4 and M7 processors do not have enough resources to implement the high-performance OPC UA server and client. With Sitara AM335x, a percentage of 53.3% useful data was obtained at a serial port speed of 12 Mb/s. As presented, Sitara AM335x consists of the ARM Cortex A8 processor and the real-time cores PRU0 and PRU1. So, the Sitara Am335x has the resources to be able to implement the OPC UA server (on the ARM Cortex A8 processor). As we have presented previously, the period of one acquisition cycle with 10 slots is 1.349 ms, so the acquisition cycle allows this speed to be used for low-level ModbusE devices. In Table 1, we can see that the time tSOPCUAR_SOPCUAA (the time between when the OPC UA server requests a ModbusE command and when the OPC UA server receives the answer) is larger than the period of an acquisition cycle. The measured values are higher at the upper level because the OPC UA client, the OPC UA server and the dispatcher use the Ethernet over Windows and Linux operating systems, which brings a delay time. ModbusE server (slave) stations can communicate useful data at high speeds among themselves without the need for these data to reach the OPC UA client. Only the values that need to be displayed reach the OPC UA client. Devices connected to the lower level on ModbusE can communicate using the publisher–subscriber pattern directly through the acquisition cycle at a speed of 12 Mb/s. Since, at the level of the PRU0 module, it was not possible to debug the software in order to see if the software for the acquisition cycle was running correctly, the PRU1 module was used to read certain variables from memory and, in this way, test if the software was working correctly. The PRU1 module was used to allow the PRU0 module to run only the acquisition cycle. All the tests were performed in laboratory conditions but not in an actual industrial process.

## 8. Conclusions

As previously presented, using the Sitara AM335x processor from Texas Instruments for an acquisition cycle consisting of 10 slots, a payload of 53.3% was obtained at a serial port speed of 12 Mb/s. The period of the acquisition cycle was 1.349 ms, so the execution time of the acquisition cycle was much lower than the time in which the OPC UA client sent and received a response to a command from the acquisition cycle. A solution to improve this time would be to use a middleware that uses the MQTT protocol instead of OPC UA. MQTT is a much simpler middleware than the OPC UA specification (over 1000 pages). As a result, the execution time between MQTT is much lower than that for the OPC UA server (which also includes the MQTT protocol). The OPC UA server is much more complex and provides a wide variety of functions and data types. Using only MQTT would allow the use of a simpler microcontroller without a Linux-type operating system.

## Figures and Tables

**Table 1:** Measured values for times tSOPCUAR_DTOUT, tDTOUT_DR(request), tDR(request)_CA, tCA_DA(answer), tDA_SOPUAA, tSOPCUAR_SOPCUAA, and tSOPCUAR_SOPCUAR.

No.	tSOPCUAR_DTOUT	tDTOUT_DR	tDR_CA	tCA_DA	tDA_SOPCUAA	tSOPCUAR_SOPCUAA	tSOPCUAR_SOPCUAR
1	3.831 ms	98.94 µs	15.6 µs	33.21 µs	240.1 µs	4.483 ms	6.129 ms
2	3.67 ms	155.8 µs	42.47 µs	71.22 µs	224.2 µs	4.73 ms	6.706 ms
3	2.961 ms	102.6 µs	159 µs	96.13 µs	231.9 µs	3.775 ms	5.402 ms
4	3.559 ms	102.7 µs	153.7 µs	68.9 µs	221.4 µs	4.328 ms	6.28 ms
5	3.352 ms	399.2 µs	34.21µs	72.52 µs	222.9 µs	4.224 ms	5.872 ms
6	3.41 ms	142.7 µs	37.56 µs	40.54 µs	232.1 µs	4.038 ms	5.766 ms
7	3.672 ms	143.8 µs	242.8 µs	64.37 µs	206.4 µs	4.515 ms	6.21 ms
8	3.508 ms	101.8 µs	244.4 µs	39.31 µs	266.2 µs	4.347 ms	5.953 ms
9	3.569 ms	140.3 µs	79.64 µs	71.35 µs	230.6 µs	4.266 ms	6.228 ms
10	3.319 ms	102 µs	88.02 µs	118.9 µs	226 µs	4.084 ms	5.716 ms

**Table 2:** Measured values for times T1 and T2.

No.	T1	T2
1	4.781 ms	5.853 ms
2	4.798 ms	5.9 ms
3	4.913 ms	5.996 ms
4	5.381 ms	6.482 ms
5	4.504 ms	5.577 ms
6	4.774 ms	6.081 ms
7	4.783 ms	5.848 ms
8	5.605 ms	6.689 ms
9	4.374 ms	5.438 ms
10	4.919 ms	5.894 ms

## Data Availability

Data are contained within the article.
